# Lewy body dementia: the impact on patients and caregivers

**DOI:** 10.1186/alzrt251

**Published:** 2014-04-25

**Authors:** Yael R Zweig, James E Galvin

**Affiliations:** 1 Departments of Neurology, Psychiatry and Population Health, Alzheimer Disease Center, New York University School of Medicine, New York, NY 10016, USA

## Abstract

Lewy body dementia (LBD) is the second most common neurodegenerative dementia in older adults, yet there remains a delay in diagnosis that limits healthcare providers’ ability to maximize therapeutic outcomes and enhance patient and caregiver quality of life. The impact of LBD on patients includes limiting the potential exposure to medications that may cause adverse outcomes, and addressing how the disease manifestations, such as autonomic features and behavior, affect quality of life. LBD impact on caregivers has been discussed to a greater degree in the literature, and there is clear evidence of caregiver burden and grief associated with disease manifestations. Other common caregiving concerns, such as access to care, prevention of hospitalization, managing behavior, and reviewing prognosis and nursing home placement, are important to comprehensively address the needs of patients with LBD and their caregivers.

## Introduction

The Lewy body dementias (LBDs) are the second most common form of dementia in older adults after Alzheimer’s disease (AD) [1,2]. LBD affects approximately 1.3 million Americans [2], although this may underestimate the true burden of disease due to the lack of timely and adequate diagnosis. LBD is an umbrella terminology that encompasses both Parkinson’s disease dementia (PDD) and dementia with Lewy bodies (DLB), with the difference being one of timing of onset between the cognitive and motor features. In this article, we will use LBD to characterize both groups unless it is specifically necessary to differentiate. LBD prevalence rates approach 5% in the elderly population and make up to 30% of all dementia cases [1,3]. Despite considerable research and defined diagnostic criteria, large autopsy series continue to highlight low diagnostic sensitivity for the clinical syndrome of LBD in patients presenting with a dementia syndrome, even among expert clinicians in specialized centers [4,5]. Many articles, including many in this special issue, address the signs, symptoms, biomarkers, pathologies, and mechanisms that are associated with LBD; all too often, however, the impact of disease on patient and family caregivers is overlooked. LBD includes a combination of cognitive, motor, autonomic, and behavioral features that when present individually or in combination often lead to delays in correct diagnosis with prescribing of medications that can have potentially serious adverse events. LBD may further cause diminished quality of life (QOL), caregiver burden, and specific care challenges for patients, caregivers, and providers distinct from those of other forms of dementia. This review will discuss the personal experience of LBD from the perspective of patients and caregivers. The material is based on the expertise of the authors, surveys of LBD caregivers, as well as a review of the literature using PubMed. The search was performed on peer-reviewed journals where the primary language was English over a 10-year period (January 2003 to December 2013).

### Delay in diagnosis

In clinical practice, patients and caregivers are not well informed about the differences between age-associated cognitive slowing, dementia as a general terminology, and the most common form of dementia - AD. It is a common misconception that memory loss is a normal part of the aging process [6]. AD affects 5.2 million adults in the United States, compared to approximately 1.3 million with LBD [2,6]. The Alzheimer’s Association has a policy and advocacy arm that lobbies for increased government funding, and the National Institute of Health and the National Institute on Aging have recently provided close to $45 million in new funding for AD research [7]. Moreover, in 2012 the National Institute of Health supported $154 million in Parkinson’s disease research [8]. While the body of evidence that comes from AD and Parkinson’s disease research theoretically has implications for LBD, the crossover potential to generalize findings may be limited due to the unique symptomatology in LBD and the competing adverse effects of medications to control cognitive, mood, motor, behavioral, and autonomic symptoms.

Despite advocacy from organizations like the Alzheimer’s Association and Lewy Body Dementia Association (LBDA), patients, caregivers, and even some clinicians may not buy into the benefits of early diagnosis and treatment of cognitive disorders [9]. LBD in comparison to AD is often poorly understood and many patients and caregivers are less knowledgeable about the disease. Physicians may know about the disease manifestations and treatment but not prognosis or community resources [10]. Even with some scientific advances in biomarkers and imaging, LBD remains largely a clinical diagnosis and therefore requires knowledge and skill by a clinician to make a formal diagnosis.

Caregivers of patients with dementia of all types express feelings of uncertainty and disorganization around the diagnostic process [11]. The LBDA conducted an online survey of LBD caregivers to better understand challenges that they face in diagnosis and day to day management of patients. Most respondents reported seeing multiple physicians over many visits before a LBD diagnosis was given, with close to 70% reporting more than three doctors were consulted. On average it took physicians four office visits to make the diagnosis, with 33% of the respondents reporting more than six office visits. The majority of survey respondents had received a diagnosis within 1 year (51%), and some even in the first month (19%), but a sizable minority of patients (31%) did not receive a diagnosis for more than 2 years [10].

One of the contributing factors to the delay in diagnosis is the wrong initial diagnosis given by clinicians. The LBD caregiver survey found that an initial diagnosis other than LBD was given in 78% of cases: Parkinson’s disease (39%), AD (26%), frontotemporal degeneration (4%), mild cognitive impairment (6%), or other unspecified dementia (12%), and primary psychiatric diagnoses (24%). The initial LBD diagnosis was made by a neurologist the majority of the time (62%), followed by psychiatrists, geriatricians, psychologists and, lastly, primary care providers. Once the diagnosis was established, around 50% of LBD patients had to see two or more clinicians for symptom management and 58% of caregivers reported difficulty with managing the care among different providers [10].

A relatively common refrain heard after an initial diagnosis when many patients return to other providers is ‘They don’t even know what Lewy body dementia is’. The LBD caregiver survey supports this, with 77% of caregivers reporting difficulty finding a physician who knew about treating LBD [10]. It has also been hypothesized that this lack of knowledge is isolating for caregivers and they are less likely to receive support from others in the community at large [12]. Twenty-five percent of LBD caregivers have reported that no one understands what they are going through and as a result 40% report feeling isolated [13]. It should be noted that this survey only included LBD caregivers and no direct comparison with other forms of dementia can be made. A follow-up survey of caregivers of patients with different forms of dementia is underway and hopefully can provide direct comparisons.

### Improving clinical diagnosis

A clinical diagnosis of LBD is largely made based on criteria established by the Consortium on DLB International Workshop for the Clinical Diagnosis of DLB [1] and the Movement Disorder Society Task Force Guidelines for PDD [14,15]. Early features of LBD help to distinguish it from other neurodegenerative and psychiatric disorders and collecting a comprehensive history from the caregiver is one of the best first steps in diagnosis. Improving clinical diagnosis is particularly important to avoid medications that can exacerbate symptoms or cause poor health outcomes and even death. Additionally, treatment options such as cholinesterase inhibitors can be therapeutically very beneficial [16]. Early diagnosis also helps to provide early caregiver support and link caregivers to needed resources in the community. One example of a tool available to improve clinical diagnosis is the Lewy Body Composite Risk score for which a cutoff score of 3 provides a sensitivity of 90% and specificity of 87% to suggest Lewy body pathology as the underlying cause of dementia [17]. The Mayo Fluctuations Questionnaire [18] and Mayo Sleep Questionnaire [19] are also helpful in evaluating for other features such as cognitive fluctuations and REM sleep behavior disorder that otherwise are difficult to quantify clinically. A comprehensive history also helps to establish autonomic dysfunction that may have been previously explained by age or other medical phenomenon.

A comparison of DLB with AD regarding early cognitive symptoms suggests that memory impairment was reported 57% of the time in the DLB compared to 99% in the AD group. The DLB group reported early visual hallucinations, depression, problem solving difficulty, gait problems, and tremor and stiffness. Of the DLB core features, hallucinations were the most common symptom prior to an initial evaluation, followed by parkinsonism and cognitive fluctuations [20]. This is consistent with other reports of extrapyramidal signs, cognitive fluctuations, hallucinations, and sleep disturbances as predictors of DLB [17].

Once a diagnosis of LBD is made, using a collaborative approach in the outpatient setting to manage patients is particularly valuable. LBD patients and caregivers use more resources in long-term residential care, and require more outpatient care, informal care, community services, and pharmacologic therapy than AD patients [2]. Social workers who function either as geriatric care managers, provide counseling, or coordinate concrete services are vital resources. In our experience, locked memory care units for AD patients who wander may not be necessary or appropriate for someone with LBD due to their physical limitations. Additionally, the behavioral disturbances common in LBD may exclude patients from many of these units, so navigating options with an expert is helpful. It is also beneficial for patients and caregivers to have a clinician easily available by phone in the outpatient setting to prevent unnecessary hospitalization, manage acute behavioral episodes, and provide guidance on symptom management.

## Impact of disease on the patient

### Exposure to medications and serious adverse events

As discussed, there may be a delay in timely diagnosis of LBD so patients with behavioral manifestations may inadvertently be treated with a typical neuroleptic or atypical neuroleptic with D2 receptor antagonism, which can worsen parkinsonism, and exacerbate other features such as sedation and orthostatic hypotension [2]. The greatest concern with the use of typical neuroleptics is neuroleptic malignant syndrome (NMS), which may be fatal. NMS is caused by central blockade of dopamine and includes muscle rigidity, hyperthermia, and autonomic instability. It can also occur from certain anti-emetics such as metoclopramide [21]. While NMS is perhaps the most serious side effect, a similar but more common adverse reaction, neuroleptic sensitivity reactions, can be seen in DLB, PD, and PDD. Neuroleptic sensitivity reactions, which can occur in 30 to 50% of DLB patients, include sedation, increased confusion, rigidity, and immobility that may occur after taking a neuroleptic medication. They are just as likely to occur in patients with mixed pathology including AD, supporting the need for accurate diagnosis [22].

Management of LBD should take into account withdrawal of medications that may potentially exacerbate parkinsonism or worsen cognition, such as medications with anticholinergic or antidopaminergic mechanisms of action [16]. This involves the careful weighing of risk versus benefit as patients with disabling tremor or autonomic features such as siallorhea or urinary incontinence may benefit from an anticholinergic medication. Behavior that interferes with patient care or safety, or safety of the care provider, likely warrants an antipsychotic agent despite the potential risks, including the black box warning of increased risk of death and/or elevated blood sugars. Parkinsonism that limits independence or mobility may benefit from carbidopa-levodopa, and the trade-off may be hallucinations or exacerbation of behavioral features. Anti-emetic drugs that may be necessary for other conditions, such as prochlorperazine, promethazine, and metoclopramide, can worsen parkinsonism. REM sleep behavior disorder that interferes with good quality sleep or puts the caregiver at risk for injury should be treated, but rare events that are not concerning may not warrant a medication such as clonazepam, which can increase risk of falls in older adults. Patients with LBD in the early stages can provide input on how bothersome a symptom may be to them, which clinicians should use to guide clinical decision making about treatment.

### Patient quality of life

QOL indicators in patients with dementia have been shown to be associated with inner strength and patients more likely to complete instrumental activities of daily living (IADL) exhibit higher levels of QOL [23]. The evidence on QOL in patients with LBD is lacking, but one study that did seek to investigate QOL found that DLB patients were less able to self-report QOL compared to AD patients with similar cognitive performance [24]. Caregivers’ assessments of patient QOL were lower than patient self-reporting. However, regardless of whether the patient or caregiver determined QOL, the DLB patients scored lower on QOL measures compared to AD patients. One possible explanation for this was the higher apathy seen in DLB. Caregivers rated DLB patients to be in a health state equal to or worse than death in 24% of cases, compared to 6% in the AD group. QOL determinants in DLB patients included the Neuropsychiatric Inventory (NPI) score, apathy, delusions, dependency with IADL, and whether the patient was living with a caregiver [24].

While each LBD patient and caregiver may report their own individual concern about which disease feature most affects QOL, autonomic symptoms and behavioral features are particularly common complaints. Constipation may precede a LBD diagnosis by more than a decade [17]. Autonomic features of LBD, especially fatigue, urinary and bowel symptoms, postural dizziness, and siallorhea are clinically very bothersome to patients, and have a detrimental effect on QOL [17,25]. Autonomic symptoms have been associated with poorer physical and social function and overall perception of general health [25].

Recurrent well-formed visual hallucinations are one of the core features of LBD, and may occur in up to 80% of patients [26]. One study found that the most common visual hallucinations included fully formed adults or children (84%), animals or insects (37%), and objects (39%) [27]. Hallucinations were more likely to occur early in the disease course, within the first 5 years of diagnosis. Some patients have reported pleasurable hallucinations [28].

Early misperceptions and family misidentification are more likely in patients with DLB, and can occur later in the disease course in those with a mixed pathology with AD [27]. Patients may exhibit hallucinations even with milder cognitive deficits, and may therefore be concerned about reporting this to family or clinicians. Capgras syndrome, a delusion that a person is replaced by an imposter with a similar appearance, is also common in DLB [29] and can be quite disturbing to patients. Patients with DLB and Capgras syndrome are more likely to have visual hallucinations and anxiety [29].

Clinically, patients with LBD may have more insight into their cognitive deficits when compared to patients with AD. When insight is retained during hallucinations, patients may be able to understand the hallucinations are not actually present and are therefore less disturbing. In addition, because episodic memory deficits can be improved with cuing, patients can use strategies to help jog their memory such as appointment books and calendars [30].

Patients with dementia want their concerns to be validated by providers [11]. Open communication helps to strengthen the relationship between the provider and patient, which is also why patients should be a part of all discussions about diagnosis and treatment planning.

## Impact of disease on family caregivers

### Caregiver burden, grief, and well-being

It is important to understand caregiver burden and overall well-being in LBD due to the repercussions chronic stress and burden have on both caregiver and patient health outcomes. The LBDA caregiver survey found that over 90% of LBD patients were unable to perform certain IADLs, such as shopping and cooking, and over 60% needed help with more basic activities of daily living. Caregiver respondents reported medium to high levels of burden, with the most frequent burden items including fear of the future for their loved one, stress related to being a caregiver with other personal responsibilities, social life interference, and uncertainty. Spousal caregivers had higher burden scores than non-spousal caregivers. The factors most likely to contribute to caregiver burden were sleep and mood disturbances [13].

When patients with DLB and AD who have similar cognitive deficits are compared, the DLB group exhibits more behavioral features with a higher frequency of hallucinations, apathy, and appetite changes based on the NPI. The DLB group caregivers endorse higher caregiver distress compared to AD caregivers, and high distress is associated with delusions, hallucinations, anxiety, and apathy. In this study, no association was reported between caregiver distress and performance on a functional scale, although the DLB group fared worse on activities of daily living and IADL scales [31].

These findings are consistent with other reports that LBD caregivers are more distressed than AD or vascular dementia caregivers [32]. More specifically, PDD caregivers were the most distressed but there was no significant difference between this group and the DLB group. Similarly, the NPI findings consistent with depression, anxiety, delusions, and apathy were associated with caregiver distress across all dementia groups, and delusions and hallucinations contributed to caregiver stress in PDD and DLB. Daytime somnolence, one feature of cognitive fluctuations seen in LBD, was also associated with caregiver stress [32].

### Access to care

Caregivers of patients with LBD are often younger than those with AD. The mean age of caregiver respondents to a survey investigating LBD caregivers was 55.9?±?12 years and was predominately female [13]. Younger spousal caregivers have unique challenges as they may still be caring for their parents and/or children in addition to their spouse. Support groups, especially in smaller cities or rural areas, may be geared towards AD or PD and therefore are not as valuable due to the specific challenges in LBD. Patients with LBD may have had to retire early due to cognitive or motor symptoms, and their financial and retirement plan may not have taken into account dementia at a younger age of onset.

Patients and caregivers with dementia report a delay in receiving needed support services due to a lack of knowledge by providers. Specialized memory centers are generally better at helping caregivers access services, but there can be a referral delay [11]. Despite clear evidence of caregiver burden, only 29% of LBD caregivers evaluated by survey reported using paid care in the home and 21% used an adult day program, with 40% reporting the need for additional help. Cost of care is a concern, although a low percentage were attending a support group (23%) or counseling (12%) [13], which are more likely to have less financial cost. The survey was accessible via the LBDA website so presumably caregivers who completed the survey may be even better informed about the disease and manifestations, yet still did not have adequate support.

### Care challenges

The multiple symptom burdens that LBD patients experience complicates the provision of care. Clinical management is simplified when clinicians focus on the symptom that is most disturbing to the patient, while incorporating input from the caregiver as well. Even more challenging is when the patient and caregiver disagree - for example, a patient with well-formed visual hallucinations may not be disturbed by this phenomenon while caregivers may rate this as a primary concern. Furthermore, caregivers may be more disturbed by cognitive fluctuations that the patient may not recognize. Autonomic features, particularly symptomatic orthostatic hypotension, constipation, urinary symptoms, and siallorhea, tend to be equally disturbing to patients and caregivers. Clear discussion about symptom management is crucial due to the paradox often present with medications for one symptom that exacerbates a separate symptom.

### Behavior

As noted, psychiatric features can be an early sign of LBD and are more likely to be present in the early stages when compared to AD [27]. LBD patients are more likely to exhibit high scores on the NPI for hallucinations, night-time behavior, and apathy. Delusions can revolve around stealing, strangers living in the home, and being in danger, and may get worse as the disease progresses [33]. One particular challenge with delusions is that they are false and ‘fixed’; therefore, the patient truly believes it to be the case and it can be difficult or impossible to convince them otherwise. They may also be centered on a loved one, so the spouse who already may be burdened also has to cope with a focused delusion. Delusions of spousal infidelity or Capgras syndrome are particularly challenging to spouses.

### Prevention of hospitalization

Management goals for all patients with dementia should include avoidance of hospitalization. This is particularly important for LBD patients if they present with behavioral manifestations due to the risk they may be given a neuroleptic and suffer adverse outcomes. As in all dementias, underlying medical illness can initially present as worsening confusion. This presentation can be challenging in LBD as it can be difficult to distinguish from the fluctuating disease course; however, reports of any acute cognitive decline should trigger a medical investigation. Patients with LBD may be more likely to have a delirium episode preceding diagnosis and have recurrent episodes of delirium when compared to AD [34].

Sixty-four percent of LBD caregivers self-reported a crisis situation had occurred in the prior year, and the most common place where help was sought was a hospital emergency room. Twelve percent of respondents additionally needed inpatient psychiatric care [13]. This again highlights the need for outpatient clinicians to be easily accessible and to utilize a collaborative approach where possible to prevent unnecessary hospitalizations.

### Surgical intervention for patients with Lewy body dementia

Surgical interventions are a common area of concern for patients and caregivers. Dementia in and of itself, and a diagnosis of LBD in particular, does not preclude necessary surgical interventions. This can become more of a gray area when optional procedures such as knee or hip replacements are considered. Clearly, arthritic joints can severely limit QOL but there is a balance when considering potential repercussions from anesthesia and the rehabilitation potential with a pre-existing cognitive and functional impairment. The relationship between general anesthesia and LBD specifically has not been robustly examined. Patients with LBD may respond poorly to certain anesthetics and experience postoperative delirium and/or functional decline [2]. Patients with Parkinson’s disease need careful oversight of their drug dosing regimen so it is impacted as little as possible pre- and postoperatively [35]. A discussion with both the surgeon and anesthesiologist prior to surgery about the LBD diagnosis is prudent.

### Prognosis and nursing home placement

Patients and families expect clear and concise information about dementia prognosis and are often disappointed with the divergent responses from clinicians. Prognosis is difficult from the clinician perspective because of the significant variability among patients and the multitude of contributing factors to health outcomes. Additionally, there is conflicting information about LBD progression, with an average duration of 5 to 7 years and range of 2 to 20 years [2]. Patients and caregivers are sometimes relieved to be given a diagnosis other than AD, due to lack of knowledge about other degenerative dementias and a sometimes false perception that anything else is preferable. Because LBD is often misdiagnosed or underdiagnosed, there may be a different sense of relief with diagnosis to finally receive an answer that explains the constellation of symptoms.

Patients with DLB, particularly men, have an increased mortality risk compared to patients with AD. One study found the median age at death among DLB patients to be 78 years, compared with 85 years for those with AD. Other contributing factors to DLB mortality include at least one *APOE4* allele, other comorbid conditions, extrapyramidal signs, and difficulty completing activities of daily living [36]. The presence of orthostatic hypotension, but not other signs of autonomic dysfunction such as constipation or urinary incontinence, has also been associated with increased mortality risk [37]. DLB patients with extrapyramidal signs and depression are more likely to enter a nursing home, although those individuals with a tremor-predominant movement disorder may be at a lower risk than those with akinetic-rigid presentations. Once in a nursing home, DLB patients have increased mortality rates compared to AD patients [36].

Other comparison studies evaluating survival differences in patients with AD and DLB have found that when comparing the groups based on Mini-Mental State Examination score, the survival time is shorter in DLB [38]. The evidence suggests that DLB patients may have worse mortality outcomes; however, the rate of cognitive decline over time is similar between DLB and AD patients [36]. There is no difference in mortality rates or time to nursing home placement between patients with pure forms of DLB compared to mixed pathology with both DLB and AD [36].

Similarly, when comparing nursing home admission (NHA) rates in patients with mild DLB and AD, men with DLB had the shortest time until NHA. The use of antipsychotic medication and the duration of symptoms prior to DLB diagnosis were associated with an increased rate of NHA, whereas use of acetylcholinesterase inhibitor had an inverse relationship with NHA. Patients with mild DLB experience durations of NHA almost 2 years shorter than those with AD [39].

While end of life prognosis can be difficult, providers can council patients and families to expect an earlier loss of independence in DLB compared to other dementias [13]. Clinically, caregivers often seek placement when care challenges such as behavioral manifestations and/or incontinence are present, both of which can be a significant area of concern in LBD. Nursing home placement is an especially difficult decision in the LBD population because often patients and caregivers have already experienced a lack of provider knowledge about the disease. There is a concern that the patient will be overmedicated, or medicated inappropriately. Providing ongoing education and utilizing information from the LBDA, such as the ‘medications to avoid’ wallet card or documenting ‘allergies’ to neuroleptics, may be useful. Staff education, especially to direct care providers in long-term care such as nurses and nursing assistants is valuable.

## Conclusion

LBD is a complex and multi-symptom disorder that diminishes patient QOL and increases caregiver burden. The impact on patients and caregivers is tremendous in that it affects not just cognition, but includes motor, behavioral, and autonomic features. In addition to coping with the disease itself, there is a lack of knowledge both in the public and private sphere, which increases feelings of isolation and lack of trust in the medical establishment. The delay in diagnosis increases healthcare cost and utilization and combined with increased rates of caregiver burden due to behavioral manifestations and loss of function further supports the need for increased awareness of the disease effects.

LBD patients have unique care challenges and there is a resultant need to always consider risk versus benefit in clinical decision making such as medication management, hospitalization and surgical intervention. The prognosis for patients in terms of mortality and nursing home placement are poorer than those with AD so clinicians must always keep the patient’s QOL in the forefront when making decisions about LBD symptom management. It is important to always consider LBD from the perspective of patients and caregivers because of the symptom burden and, without a cure at this time, management must be individualized to each patient to maximize outcomes.

## Note

This article is part of a series on *Lewy Body Dementia*, edited by Ian McKeith and James Galvin. Other articles in this series can be found at http://alzres.com/series/LewyBodyDementia.

## Abbreviations

AD: Alzheimer’s disease; DLB: Dementia with Lewy bodies; IADL: Instrumental activities of daily living; LBD: Lewy body dementia; LBDA: Lewy Body Dementia Association; NHA: Nursing home admission; NMS: Neuroleptic malignant syndrome; NPI: Neuropsychiatric inventory; PDD: Parkinson’s disease dementia; QOL: Quality of life.

## Competing interests

The authors declare that they have no competing interests.
